# Performing food and nutritional security in Europe: claims, promises and limitations

**DOI:** 10.1007/s12571-018-0853-9

**Published:** 2018-11-28

**Authors:** Paul Hebinck, Henk Oostindie

**Affiliations:** 1 Sociology of Development and Change, Wageningen University, Wageningen, The Netherlands; 2 Department of Agricultural Economics and Extension, University of Fort Hare, Alice, South Africa; 3 Rural Sociology, Wageningen University, Wageningen, The Netherlands

**Keywords:** Food and Nutritional Security (FNS), Food assemblages, Promising solutions

## Abstract

Food and Nutritional Security (FNS) is attracting growing attention amongst food system scholars. Drawing on assemblage thinking, this article aims to contribute to the ongoing scholarly and policy debate on tackling food problems and societal concerns about the future and direction of the global food system. We make use of assemblage theory and the food and agrarian sociology literature to identify cases for further scrutiny. The cases were selected based on claims and discourses as well as on what is actually happening in everyday reality. The case material has been developed in the context of a collaborative research project on issues of FNS in Europe. These cases are treated conceptually as heterogeneous and fluid socio-material constellations that contain promises to address different aspects of contemporary FNS challenges. An unravelling and unpacking of FNS inspired by assemblage theory enriches the food security debate through focussing on real, performed, new and promising configurations, and their underlying governance arrangements.

## Introduction

Critical food systems analysts (Clapp 2016; Lang 2010; Lang and Barling 2012; McMichael 2009, 2013; van der Ploeg 2010, 2016; http://www.foodsystemsacademy.org.uk) point out that dramatic changes at the level of food systems have occurred roughly since the late 1950s and early 1960s. The ever-increasing ecological footprint of our food, the continuous squeeze on agriculture, the increasing reliance on external resources, the corporatisation of the production, processing, retailing and marketing of food and the increasing role of agro-food science in determining food quality are processes that have contributed to a gradual (re)shaping of our food system. We cannot ignore that the system increasingly experiences systemic shocks and stresses from which it does not always recover well, exacerbating existing vulnerabilities as well as generating new ones. The continuity of food and nutritional insecurity situations are proof of the severity and permanence of these shocks and stresses, as are the global food riots arising as a consequence of food price hikes, food poverty, famines, food-related diseases and food quality issues. That such situations endure raises serious questions about the manageability of the food system and whether and how the current food system can be re-designed such that these stresses and shocks reduce in size, scale and intensity.

This paper explores how, in many different ways and modalities, food security issues are being handled at the moment in Europe. Our entry point is that next to the need to understand why and how food questions emerge, we find, like Piatti and Dwiartama (2016) and Carolan (2016, 2013b) that it is important to investigate empirically how food security is performed in given contexts, and what claims are made with regard to impact and reach. We set out to answer these question by drawing on case material developed in the EU-funded project that set out to document the variety of ways in which food and nutritional issues in Europe are being addressed, and to provide inputs into policy debates on how these are to be addressed in the future. We investigate these ways of performing food security as real, existing, human and non-human actor configurations. They are analysed in this article as *assemblages* that set out to perform food and nutritional security (FNS) in diverse ways (see also Dwiartama and Piatti 2016; Carolan 2016). These represent promising practices, performing food security in ways that potentially out-perform mainstream ways of attending to FNS questions. They have unfolded over time as they have been enacted and given shape by social actors who sought to address issues and concerns associated with FNS. They do so from a variety of ideological backgrounds. The assemblages, as we shall show, vary in orientation, content, intensity, scale and degree of success, but all share the intention to *purposively* attempt to reassemble and reconfigure the food system or parts thereof to address societal FNS concerns. The assemblages we focus on in this article all materialised as concrete responses to the different types of FNS vulnerabilities associated with an erosion of entitlements to food that emerged in the wake of the – gradual but definitive – retreat of the welfare state as part of neo-liberalisation and globalisation tendencies.

Investigating and theorising the food security performance through an assemblage prism adds substantial value to current food security analyses. Firstly, since assemblages are fluid, dynamic places where human and non-human actors (re)connect in novel ways, exploring their performance and discursive framing enables one to trace societal dynamics that address food-related issues in ways that are unexpected*,* hidden from the public eye and/or marginal and undocumented. As such this may provide more comprehensive insights into FNS promises and innovations, how these concretely materialise and what their legitimising discourses are and may in turn stimulate debate on how viable and sustainable FNS alternatives might be. Secondly, an assemblage perspective enables one to acknowledge the relevance of new elements that are brought into the debates about food security, such as health and public entitlement efforts. Thirdly, it advances, in a nuanced, analytically innovative way, the much-needed move to go beyond primarily technical or production foci on FNS (Devereux and Maxwell 2001; Allen 2013; Marsden 2013, 2017; Fouilleux et al. 2017) and to reflexively interrogate the role of public policy-making and governance arrangements (Lang and Barling 2012; Duncan 2015) by engaging critically with various strands of both political economy and governance scholarly thought (Stoker 1998).

In the following sections we will further substantiate the added value of theorising food system dynamics from an assemblage perspective, starting with further elaborating on what energises food assemblages and what guides them, discursively and practically, in dealing with FNS concerns. We then explain how we selected three rather distinct FNS assembling practices in condensed form. The fourth section interlinks the added value of combined theoretical and empirical insights to ongoing scholarly debate on food governance challenges. This is followed by some concluding remarks.

## Positioning the paper: structural transformations or a perfect storm?

It is widely recognised in the literature and in policy-making and practitioner circles that the range of food policies and interventions responding to the transformations of the globalising food system have not effectively and adequately addressed, let alone solved, the questions of development and food security (Marsden 2013, 2017; Wright and Middendorf 2008; Patel 2007). There is consensus that the twenty-first century ‘is the worst of times because more people go hungry than at any point in human history’ Allen (2013: 135). It gets worse when we realise that there is no agreement on how to tackle the problem. The idea and promise that a ‘simple’ technological fix can and will sustainably save the world from hunger are, however, still pervasive in many academic, policy and corporate investment discourses. The current Sustainable Development Goals are testimony to that. There are plenty of suggestions that production and productivity increases and sustainability can be simultaneously achieved through ‘sustainable intensification’ (Hunter et al. 2017; Garnett et al. 2013). Propelled by an extended neo-liberalisation of the economy (Allen 2013; Marsden 2013; Duncan and Barling 2012) and the agro-industrialisation and globalisation of food systems that is driven by the global food companies (van der Ploeg 2010, 2016; McMichael 2009, 2013, 2014; Fouilleux et al. 2017), technical engineering has evolved as a major solution-oriented discourse (Scott 1998). The advances and directions in (agro)food and nutrition science, biochemistry, technical agronomy and plant breeding serve as perfect seedbeds for the further nurturing of such an engineering and production discourse.

The claim that the stresses and shocks of our food system can be solved through management, the application of modern scientific insights (e.g. crop genetics, genetically modified organisms, precision farming) and creating positive and productive policy environments is, however, severely and increasingly questioned in the academic literature (Scott 1998; Leach and Scoones 2005; van der Ploeg 2010; Clapp 2016). Together with activists of the food sovereignty-related social movements (Aistara 2012; Levkoe 2012; Martínez-Torres and Rosset 2014; Borras et al. 2015) it is argued that a further deepening and expansion of the *agro-ecological foundations of food systems* will generate a more equitable and sustainable food system capable of tackling some of the world’s major food questions. This opinion is to a certain extent also captured in the *sustainable intensification of agriculture* position (Baulcombe et al. 2009; Garnett et al. 2013). Central to the idea of sustainable intensification is to achieve levels of intensified land use and use of other material and non-material resources such that the pressure on their use is reduced or limited; this, in turn, would underpin and guarantee sustainability. Equally, food production in a context of population growth must ultimately be sustainably increased if it is to continue to feed people in the future (Garnett and Godfray 2012; Hunter et al. 2017). This position resonates widely in many policy circles, international fora, academia and (agro-)food expert networks (Garnett et al. 2013). However, when it comes to defining *what* intensification is, *how* to intensify, *what* is to be intensified and *what* the nature of the knowledge is that is required to intensify and enrich resources sustainably, controversies emerge (Wezel et al. 2015; Tittonell 2014; Caron et al. 2014). The direction of food systems and with what means intensification processes should proceed remains contested. The difference between what we could call intensification sciences and agro-ecology becomes rather clear. Modern science (managed and controlled by science institutions and agribusiness) is, for many intensification protagonists, positioned as a key ingredient for FNS in the context of a growing world population. In contrast, agro-ecology sciences revolve around local, agro-ecological knowledge that is produced and controlled by producers and consumer communities. These views and positions are not so easy to merge and perhaps cannot be merged.

Moreover, there is little agreement about what processes lead to recurrent food crises. The 2008 food crisis is understood as the outcome of a ‘perfect storm’ (De Gorter et al. 2013a, b; Headey et al. 2010; Headey and Fan 2008, http://www.globalissues.org/news/2010/11/19/7694) – as the result of an unfortunate combination of circumstances. For some observers and agencies such as the World Bank, the International Food Policy Research Institute and the Food and Agriculture Organisation the food crisis is perceived as interconnected with many other issues and processes (i.e. climate change; security situations; failing states; harvest failures; high energy prices, notably for oil; increasing demand for biofuels and flex crops). Food crises are contingent events that will only end when food prices decline, energy prices reduce and investments in a second (or third) green revolution materialise. Targeted policies and tax measures will have to balance the potentially competing demands on land for urban use and those for the production of food, fodder and fuel. There is, in other words, a strong belief in the potential of *governing* the food question. In stark contrast to the perfect storm analyses and analysts, critical observers perceive food crises and questions as outcomes of built-in structural processes: an unprecedented industrialisation of agriculture, liberalisation of the world food market, the rise of food empires and failing agrarian policies (Rosin et al. 2011; van der Ploeg 2010; Lang 2010; McMichael 2009; Jarosz 2009, 2014; Clapp and Helleiner 2012; Magdoff and Tokar 2009).

The complexity of the food question has been substantially increased, and to a degree exacerbated, by the emergence of new actor groups and configurations that play a role in reframing and reshaping the fundamentals of the global food system. They include, not just supermarkets and global food conglomerates, new food cultures and new eating habits, but also new actor constellations that target issues of food poverty and other critical aspects of our globalising food system. These involve civil society groups (e.g. church-based and urban-based groups, food consumer movements, producer organisations) but also public–private partnerships (e.g. food banks; school feeding projects) and local governments (implementing municipal- or country-level food policies). Each of these actor groups frame the food question in their own way and construct managerial discourses of efficiency and modernity that mix with or at times conflict with newly emerging discourses of redistribution through food assistance and food relief, food sovereignty and food justice. Their new discourses and associated practices add to the existing complexity of dealing with food-related issues.

This paper documents some of these newly emerging dynamics around food as assemblages and what they actually entail, what claims are made, at what scale they operate and what they aim to achieve. Some of them get attention in the media and in the food studies literature, where they are often referred to as ‘alternative food networks’ (Hodgins and Fraser 2018; Le Velly and Dufeu 2016; Phillips 2016, Wald and Hill 2016; Wiskerke 2009) while others remain rather obscure and undocumented.

## Conceptual framing and methodology

Our point of departure is that food systems are not uniform but heterogeneous in that there is not one – neither ideal nor optimal – route to achieve the objectives of FNS in Europe or elsewhere in the world. Food systems are constituted by various co-existing and interacting, but often contrasting, food practices. Along with Dwiartama and Piatti (2016) and Rosin et al. (2013), we conceptualise these practices as assemblages. These emerge from and are shaped by divergent social values and norms, by discourses, by the way in which human and non-human resources are connected as well as by the historical, social, cultural and political contexts in which they are embedded. Assemblages are coordinated by human actors in search of some semblance of certainty to realise objectives (Rosin et al. 2013). Assemblages are not just theoretical constructs requiring an assemblage methodological framing and positioning, they are also, simultaneously, unfolding heterogeneous, dynamic, multi-layered and complex practices entailing the mutual shaping of the socio-cultural, institutional and biotic elements that constitute the assemblage. Through manifold interconnections, (food) assemblages continuously generate new assemblages with ever different and new attributes. This conforms with the original ideas and thoughts of Deleuze and Guattari (1987) about assemblage as continuously transforming and/or reproducing. An assemblage perspective has gained substantial ground in social sciences investigating development processes as non-linear, fluid and emergent and not necessarily patterned by hegemonic forces. Insights from assemblage theory are applied to a variety of themes and sub-fields such as forestry management (Li 2007), land issues (Li 2014), urban spaces (McFarlane 2009; Anderson and McFarlane 2011; McFarlane and Anderson 2011), food problems (Rosin et al. 2013), planned interventions (Umans and Arce 2014; Kimanthi and Hebinck 2018), regional development (Woods 2015; Pasmans and Hebinck 2017) and environmental governance (Forney et al. 2018). It is useful to briefly discuss the processes that define and simultaneously produce various new types of assemblage. Assemblages are subject to processes of territorialisation (becoming stable, coherent and solid) and de-territorialisation (becoming unstable and fluid). For Deleuze and Guattari (1987) processes of ‘deterritorialisation’ and ‘territorialisation’ are central to depict the dynamics of change and transformation. Nail (2017: 34) summarises four kinds of deterritorialisation process:

(1) ‘relatively negative’ processes that change an assemblage in order to maintain and reproduce a new and established assemblage; (2) ‘relative positive’ processes that do not reproduce an established assemblage, but do not yet contribute to or create a new assemblage—they are ambiguous; (3) ‘absolute negative’ processes that do not support any assemblage, but undermine them all; and (4) ‘absolutely positive’ processes that do not reproduce an established assemblage, but instead create a new one.

We return to this typology when it comes to selecting case studies for further analysis. The reasoning behind these typologies leads us to define assemblages as Li (2007: 265) does: the ‘*grafting of new elements and reworking old ones; employing existing discourses to new ends*’. Such a definition emphasises the capacity of actors to reassemble their food system, both socially and materially. This conceptualisation provides an avenue for exploring how food, and also the meaning and the making of food, is continuously reconfigured and how the socio-material infrastructures, the social actors and the relationships between them change in such a way that previously existing elements and interlinkages are rearranged to form new connections and relationships that did not exist previously (Li 2007; Anderson and McFarlane 2011). These assemblages establish ‘alternative’ routines and new patterns of connecting or reconnecting FNS resources, leading to new routines and new social relationships. Hargreaves et al. (2013) theorise that the reconnecting or reassembling of FNS resources mostly cuts across multiple regimes (e.g. the food regime, the transport regime, the energy regime, the policy regime, and so on). Hence innovations, or rather the crafting of new elements, are perceived as ‘regime-crossing systems of practice’ allowing us in turn to approach new food practices and initiatives as specific responses to food system transformations and – more generally – as expressions of agency (Long 2001) that are displayed by actors in the processes of (re)constructing assemblages, whether territorialising or deterritorialising.

While pursuing such analysis we need to take Allen’s (2011: 154) warning on board that

the task is not so much one of pinning down the ‘correct’ definition of assemblage or simply declaring a certain fidelity to Deleuze’s vocabulary, as it is one of exploring what avenues of enquiry are opened up and what questions are made possible by thinking through social and material formations as assemblages*.*

Assemblages allow avenues of enquiry that fit with the fluidity, contingency and nonlinearity of the processes of change that accompany contemporary FNS concerns. It specifically allows us to move beyond problematic binaries such as ‘internal’ versus ‘external’, ‘new’ versus ‘old’, ‘alternative’ versus ‘hegemonic’, ‘consumptive’ versus ‘productive’ or ‘local’ versus ‘global’. McFarlane (2009: 562, quoting Ong and Collier 2005) notes that ‘*in relation to the “global”, the assemblage is not a “locality” to which broader forces are counterposed. Nor is it the structural effect and outcome of such forces*. *An assemblage is the product of multiple determinations that are not reducible to one single logic*’. This implies that the logic and dynamics of assemblages cannot only and exclusively be understood by referring to the workings of capital, capitalism and the capitalist food system and its development over time. In the development sociology and anthropology literature this point of non-linearity has been made forcefully (Olivier de Sardan 2006; Long 2001). The relevance of assemblage theory is that it goes beyond understanding development as linear and explicable as structural processes.

The temporality of an assemblage is thus to be treated as *emergent*. It does not always involve new forms, but forms that are shifting, in formation, or at stake. In a similar vein*,* Levkoe and Wakefield (2014: 306*)* stress that ‘*assemblages are difficult to bound because each component of the system is connected in myriad ways to other components ad infinitum; boundaries, therefore, become delineations of convenience rather than absolute, fixed borders between “in” and “out”*’*.* Relevant for our purpose is that assemblages bring together ‘*diverse interests […] without ideological coherence as a necessary precondition*’ (ibid: 315) and that ‘*theorising networks as complex assemblages help to visualise and understand that initiatives with diverse goals and approaches can work together without ideological coherence*’ (ibid: 317). McFarlane (2009: 567) similarly concludes that assemblages are ‘*resultant formations, placing agency less in the realm of direct causes and more in the realm of sources, which come together in particular events*’. Bennet (2005: 459) further elaborates on the composite and distributive nature of the agency of assemblages by arguing that

[e]mergent causality is another way of conceiving a nonlinear, indirect causality, where instead of an effect obedient to a determinant, one finds circuits where effect and cause alternate position and redound back upon each other. If efficient causality seeks to rank the actants involved, treating some as external causes and others as external effects, emergent causality places the focus on the process as itself .

### Methodology

Significant for selecting and identifying the cases for further scrutiny are:

1. Assemblages that are *discursively framed*. The discourse centres on societal concerns and debates about (newly emerging) food-related *vulnerabilities* that require action. The framing usually manifests in terms of *claims;* the key social actors make a claim that their assemblage is performing FNS and thus contributing to a sustainable FNS landscape. One can thus distinguish between, and recognise assemblages by their discourses.
2. Assemblages that are actively *performed.* While responding to societal concerns and debates (such as food poverty), the food system is actively reconfigured or minor adjustments are made. Assemblages are ‘work in progress’, not yet completely finished, continuously evolving and adjusting to changing contexts (e.g. pricing, taxes, policies, consumption practices, food culture). They are in a continuous process of assembling, reassembling and transformation.

#### Selection of cases

We take our case material from the TRANSMANGO project which was funded through an EU grant. Our research programme was not primarily interested in studying either (re)assembling processes with leading roles for global, corporate food enterprises^1^ or the attempts to rebuild our food system through technological fixing.^2^ Instead, and from the start, the project purposefully focussed on reassembling practices that hold alternative promises for solving some of the pressing food questions in relation to food accessibility, affordability and health. Framing it in terms of Deleuze and Guattari (1987), we were specifically interested in the ‘relatively positive’ and ‘absolute positive’ contributing to and creating new assemblages. We found that reassembling processes are discursively formed and framed by two major debates and positions in the broader food security and food sociology literature.

The first debate builds on the influential arguments developed by Sen (1981, 1990). Sen, in short, argues that food security problems flow from the erosion of people’s entitlement to food. Sen (1981: 2) categorises four entitlements: ownership through commodity exchange (*trade-based entitlement*), the right to own what one grows on the farm (*production-based entitlement*), the sale of one’s labour power for purposes of earning an income so as to purchase food (*own-labour entitlement*), and the right to own what is given by others (*inheritance and transfer entitlement*). Among Sen’s strongest tenets is the assertion that food insecurity can exist without any (substantial) decline in the general supply of food and, even when food shortages are widespread, they do not affect everyone uniformly. When one or all of these entitlements erode, food insecurity and poverty can occur even in conditions of plenty (see also Gore 1993; Allen 1999; Devereux and Maxwell 2001). Processes of globalisation and deepened neo-liberalisation of the economy evoking particular hotspots of change (including loss of entitlements) produce food and nutritional inequalities across the globe, not just along the North–South divide (Dowler 2008). Globalisation processes have also turned food insecurity into a global problem. Particular food banks and related attempts to reassemble the food system refer in their writing and speech to ‘food poverty’, which requires redistribution and some form of charity. However, different groups and individuals across the globe have different commanding powers and an overall food shortage only brings out these contrasting endowments. In recognition of variability in endowment, the ‘entitlements approach’ advocates a greater refinement of the categories of those affected or not affected by food shortages (Sen 1981: 156).

The second debate revolves around the argument that the transformation of our food system gradually but increasingly disconnected food quality, health and sustainability. The ecological footprint has significantly enlarged the social and cultural distance between sites of production and those of consumption; the origin of food is increasingly unknown. These processes are strongly associated with the ongoing industrialisation of food production which simultaneously entails an ever-growing distancing from nature and makes the food system more vulnerable to shocks such as price hikes for energy, transport and processing (van der Ploeg 2010, 2016; Lang 2010; Rosin et al. 2011). The combined effect of an agro-industrialising and globalising food system is a ‘squeeze’ on agriculture (Marsden 2003) that narrows the gap between product value and costs, which threatens the sustainability and continuity of the agricultural sector, farm enterprises and the future of farmers’ families (van der Ploeg 2010). A major implication of a globalising food system is that food quality is largely defined by globalising corporate interests as well as by (agro-)food science (Dowler 2008; Ponte and Gibbon 2005). These processes of food system transformation have generated assemblages that are documented as ‘alternative food networks’. Common to their discourse is that food has increasingly become a global commodity challenging both global citizens’ rights and their entitlements to food, as a result of which food justice has become a major slogan in food sociology literature (Dowler 2008; Allen 2008). Food as a global commodity hinges increasingly on a global sourcing of food and raw material resulting in a structural disconnect from the immediate rural environment of consumers.

These two debates inspired many actor groups to begin to act on, and look for ways to reassemble and reconfigure the food system so as to respond to their food issues and concerns (Hebinck et al. 2015). To a large extent, an entitlement lens inspired the constitution of assemblages that promise to solve part of the world’s food problems by providing access to food and removing barriers to strengthen production entitlements. We understand these as a re-assembling hingeing on the ‘*re-enforcing of food consumption* and *production-based entitlements’,* notably of traditional and newly emerging vulnerable groups that make use of redistributive discourses to tackle food poverty and other forms of social exclusion. The health and sustainability inspired reassembling processes we labelled as ‘*re-connecting sustainability and health*’, often with public procurement as an important component. It is important to notice that the cases were classified into these three reassembling categories in retrospect; initially they were selected primarily on the basis of involved stakeholders’ interest in joining a participatory, fuzzy, cognitive-mapping-inspired food security scenario. Overall, the applied methodology comprised a mixed-method approach that combined this multi-stakeholder fuzzy cognitive mapping with secondary data-source analysis, stakeholder interviewing, and – in some cases – feedback sessions with stakeholders to discuss, share and complement overall case-study findings (Hebinck et al. 2015; Lord and Vervoort 2017).

## Ongoing reassembling of food and nutritional security

Below we briefly present what most significantly characterises the ongoing reassembling of food systems, and who or what drives the reassembling.

### Re-enforcing food consumption-based entitlements

The case material on Dutch food banks, food assistance in Tuscany and Bia Food in the UK all represent assemblages that aim to tackle food poverty in relatively high-income countries through the provision of food assistance. A common characteristic is that they are embedded in policy settings that are shaped by a relatively prolonged period of neo-liberalism, prolonged periods of public austerity measures and growth in structural unemployment as a consequence of deepening processes of social fragmentation and marginalisation. This is partly reinforced by the emergence of new vulnerable groups such as unemployed labourers and refugee migrants. In these settings, food assistance practices turn out to depend primarily on voluntary and charity sectors. Reassembling the food system is, in specific ways, interwoven with preventing food waste and – albeit more incidentally – with reconnecting sustainability and health concerns and active attempts to foster new types of urban-rural relations.

#### Dutch food bank practices (Hebinck and Villarreal 2016; Hebinck et al. 2018)

Food banks in the Netherlands rely completely on volunteers. They position themselves as a response to public-policy negligence of food production and income poverty concerns. Food banks are rather diverse; they profile themselves politically in dissimilar ways; they formulate their ambitions differently, employ varying food poverty selection approaches and access sources of funding in their own various ways. The national food bank association (Vereniging Nederlandse Voedselbanken, or VNV) interlinks food assistance primarily with food waste/management by establishing close relationships with retailers and food manufacturers. A professional and efficient transport system operates for the redistribution of collected food surpluses, which aligns as much as possible with individual food bank needs and preferences. Additionally, it develops projects for the processing of surplus food so as to extend possible periods of use of most perishable products and to better deal with peak surplus flows. However, this dependence on surplus food makes the national food bank association’s contribution to healthy diets questionable and subject to debate. For that reason, some local food banks are looking increasingly for more healthy food-sourcing opportunities, including establishing relations with different kinds of urban food initiative (see Section 4.2.; see also van der Horst et al. 2014).

#### Food assistance in Tuscany (Arcuri et al. 2016; Hebinck et al. 2018)

Food assistance assembling in Tuscany is, for various reasons, more complex than in the Netherlands. With a long and strong tradition of charity, food assistance entails a more diverse set of practices and actors, partly also due to more active public policy engagement. The enactment of the Good Samaritan Law in 2003, for instance, facilitated the shift of responsibility for food safety during its conservation, transportation and storage to charity organisations. This simplified the donation procedures for private firms. Different types of assistance materialised, ranging from the traditional ‘soup kitchens’ or ‘canteens’ – including smaller-scale variants with a greater emphasis on social interaction – to the ‘Emporia of Solidarity’ supermarkets where designated people can shop for free. Access to these small supermarket-like shops is controlled. Payment is with a pre-loaded electronic card, which contains a certain number of points that are in accordance with the needs of the individual. Beneficiaries of these free supermarkets include the ‘new poor’, selected on the basis of means tests by public counselling centres; these tests include various economic, social and medical criteria intended to provide a safety net for those individuals and families who find themselves in a temporary state of need. Different from the Dutch and Irish food assistance settings (see above and below), the ‘Emporia’ in Tuscany also provide fresh produce such as fruits and vegetables thanks to formal and informal arrangements with ARTEA, the Regional Agency for Payments for Agriculture, and regional fruit producers. ARTEA financially compensates regional fruit and vegetable producers for surplus production that may be delivered to Caritas, an organisation that acts a collection centre. The Emporia supermarkets complement their fresh food assortment by purchasing at food markets. They offer a range of non-food related services (e.g. social loans, microcredit services, family budget courses, Italian language courses, cooking classes).

#### Bia Food Initiative (Carroll and O’Connor 2016; Hebinck et al. 2018)

Irish Bia Food (BF) is a food redistribution charity operating as an intermediary between food companies and charities that serve disadvantaged communities. As a social enterprise, BF cooperates closely with retailers (especially Tesco) in optimising the logistics of food waste redistribution. Of the approximately 50,000 t of recoverable Irish food waste per year, BF aims to redistribute 10,000 t. The use of technology and online services are key in their operations, allowing for more tailored distribution of food products as food banks can ‘request’ foods available in the warehouse. BFI recently won the contract to administer Irelands’ wedge of the Fund for European Aid to the Most Deprived (FEAD). However, to qualify for this, BF had to branch out and establish operations in other cities. Although this was done successfully, it has left BF financially over-extended in the short term. A more recent development, connected to BF, is Food Cloud, a mobile phone app that facilitates surplus food redistribution directly from supermarkets. The Food Cloud initiative was selected as a finalist at a national competition for social enterprises. Similar to ongoing Dutch and Italian food assistance reassembling, BF does not have the authority to discriminate between healthy and unhealthy food when accepting donations. Rather, it focusses on the environmental burden of food waste and avoids active engagement in the rhetoric of food poverty.

### Re-enforcing production-based entitlements

The case materials on land access in the metropolitan area of Rome and peri-urban agriculture in Valencia embrace reassembling practices that connect human and non-human resources to strengthen urban-based food production through securing access to (peri-)urban land. Rome and Valencia are metropolitan areas that are known for their relatively high levels of urban and youth unemployment – a legacy of enduring economic crises. Yet, this only partly explains the emergence of urban environmental movements, built in part on the ambitions of younger people – some university graduates – to become farmers. Another key component of ongoing reassemblages is the availability of (semi-)abandoned urban land and the presence of other urban dwellers who discursively and actively aspire to be involved in (peri-urban) agriculture. There is clearly a close connection between the two groups, and what further bonds their reassembling is social struggle. This struggle is primarily about getting access to land in urban and rural areas and much less – certainly in comparison to previous reassembling practices – about voluntary work and charity.

#### Land access in the metropolitan area of Rome (Grando et al. 2016)

The ‘Eternal City’, Rome, passed two land access acts (Decree of Liberations and Decree Terre Vive) in the last decade. Although primarily motivated by economic issues, these acts opened up new opportunities for young farmers. They capitalised on the growing awareness that relatively large tracts of unutilised public land could be used more productively. This unutilised land is part of a masterplan, which prohibits construction in environmentally sensitive areas, on the premise that compensation areas are available to construction companies elsewhere. Since the latter category of land is not easily available, large tracts of land are available for agricultural and other activities. Particularly since the economic crises these public green spaces have become seedbeds for urban-based reassembling practices. Sometimes these are given hands and feet by cooperatives initiated by young farmers who have successfully applied for formal land entitlements through various tender procedures. The dynamics within this would-be farmer movement with (peri-)urban roots demonstrates how land access, as another, primarily production-oriented, re-enforcement of food entitlements might be imbued with social struggle by (and within) newly emerging urban food movements. The Roman case shows that this social struggle conforms with contested claims on how urban land entitlement interventions may contribute to FNS in the greater Roman metropolitan area.

#### Peri-urban agriculture in Valencia (Cerrada-Serra et al. 2016)

The peri-urban area of the Valencian metropole is characterised by the natural region of the Huerta, which comprises a complex and unique ecosystem that goes back to medieval Muslim irrigation systems (*acequias*). The Huerta faces several concurrent processes that may threaten its future: a decrease in cultivated land, pollution, infrastructural plans, urban sprawl, the abandonment of material heritage, and so on. The last decade has seen a proliferation of agriculture and food-related reassembling in the Huerta with – again – a prominent role for political struggle around younger inhabitants’ access to land. Clearly different from the Roman situation, Valencian governmental bodies have not really catered to their needs in any formal way, although bilateral, informal support relationships are emerging. Ongoing reassembling is constituted by a group of people with diverse characteristics: some have urban, others rural roots; some have access to prominent idle land resources owned by family members; many are familiar with traditional farming practices; many have access to traditional and novel emerging market channels and – most importantly – all are inspired by strongly agro-ecology and food sovereignty inspired food security ideas. Their engagement in political and organisational struggles to preserve and revalorise the unique Huerta ecosystem is therefore key for its continuity.

### Reconnecting sustainability and health

These reassembling practices are represented by the Cork Food Policy Council; Sustainable Food Cities Network, Wales; Community Supported Agriculture/Voedselteams in Belgium and the Dutch Urban Food Movement. They are practices that have emerged in settings with a relatively high purchasing power, with expanding cities, active environmental and food movements (addressing food safety and different types of quality concerns regarding the negative externalities of agri-industrial farming), and with neo-liberal and fragmented food policies. Active consumer-citizen commitment has increasingly become a regular feature in these settings, albeit it expressed in rather different ways for historically rooted reasons, such as more or less active food-market intervention traditions and food import dependencies. Consequently, in these particular settings, actors turn out to be motivated by different types of FNS concern and may have rather different expectations about how public procurement might alleviate these concerns.

#### Cork Food Policy Council (Carroll and O’Connor 2016)

Cork City has a reputation as a ‘rebel county’ in Ireland, which in relation to FNS is associated with a strongly present tradition of ‘do it yourself’, a spatial clustering of organic farms, a disproportionately high number of small-scale artisanal food producers, and a significant number of non-national food producers. It also hosts a monthly lecture series, film screenings and flash/street feasts. As the alternative food capital of Ireland, it also hosts a covered English Market and key personalities for championing high-quality local food. Its current position as a ‘foodie’ hub is further reflected in the Cork Food Policy Council (CFPC), which consists primarily of volunteer food-system experts from varied backgrounds, such as academics, grocers, food processors, farmers, gardeners, restaurateurs, and so on. Their main objective is to advocate a food system that is more sustainable, healthier and more socially just. One element explicitly mentioned is to not get ‘bogged down with academic discourse’ (Carroll and O’Connor 2016: 17) and instead search for social engagement as a way to advocate for a food-system change. Until now the CFPC has only received ad hoc funding, one of their main challenges, which they attempt to address by raising funds at the supra-national level. At the municipal level, there are often competing claims for funding, which increases the financial pressure on the CFPC. A fruitful connection has been made with the Sustainable Food Cities Network in the UK (see below) and they hope to make further international connections to strengthen their activities and impact. The activities organised by the CFPC include the construction of planter boxes for citizens and weekly maintenance and care for plants in the neighbourhood.

#### Sustainable Food Cities in Wales (Moragues-Faus 2017)

Sustainable Food Cities (SFC) in the UK was initiated by the Soil Association, Sustain, and Food Matters. These three national civil society organisations promoted the creation of multi-stakeholder partnerships at the city level. The 47 SFC members work to advance healthy and sustainable food in their localities. The main focus of their collaboration is on networking and knowledge dissemination practices about key food challenges, as well as practical solutions and best practices. Through the SFC website, experiences are shared and information and knowledge on how to develop charters, partnerships, action plans and the like are communicated. Currently the main activities of the SFC are: 1) providing a communication platform; 2) organising networking events and campaigns; 3) sharing experiences and training and 4) the funding of six Sustainable Food City Officers, selected by a national tender system that challenges City administrations to elaborate novel and promising urban food strategies. The Sustainable Food Cities Award is just one illustration of how SFC succeeds in tapping the locus of power within cities to work on upscaling, consolidating and establishing urban food strategies as novel contributors to and proponents of FNS. While food security is not explicitly part of the jargon, SFC (and individual cities) do enhance FNS outcomes – particularly the access, utilisation, and sustainability dimensions. Within the range of activities carried out by SFC, there is strong emphasis on health and sustainability. Both are placed at the centre of ongoing reassembling practices, which may further address the reinforcement of food entitlements, mostly framed as the reduction of food poverty among vulnerable groups and the promotion of better governance practices in order to effect change at the structural level. As such, SFC embraces a broad spectrum of activities and actors in its attempts to reconnect food production and consumption.

#### Community Supported Agriculture/Voedselteams in Belgium (Zwart et al. 2016)

Community-led FNS reassembling in Belgium hinges on co-creating and co-learning new ways of food production, distribution and consumption. As part of Belgium’s alternative food movement, it intends to reshape conventional market functions as 1) buying and selling; 2) storing; 3) transportation; 4) processing; 5) standardisation; 6) financing; 7) risk bearing and 8) marketing intelligence. This reshaping of market functions contributes to some degree in overcoming FNS concerns, although with variations regarding environmental, social, ethical, and health-related aspects as these turn out to be valued rather differently among involved stakeholders. Flemish Voedselteams and Community Supported Agriculture exemplify how newly emerging food-related social enterprises might entail specific promises but are simultaneously characterised by typical assemblage features such as temporality and fluidity.

#### Dutch Urban Food Movement (Hebinck and Villarreal 2016)

In the Netherlands, we find, for the most part, a relatively young urban food movement (UFM). The city of Rotterdam – whose port is an important hub for globalised food supply chains – has a relatively long UFM history due to historical, cultural, and urban planning features. In contrast, Eindhoven, the birthplace of Phillips and a high-tech centre in the Netherlands, is characterised by a novel and fragmented UFM landscape. Both demonstrate the growing interest in and commitment to food issues on the part of Dutch citizen-consumers and both showcase a plethora of urban responses to sustainability, public health, and wider urban quality of life concerns. Starting from diverse ideological angles and sustainability perspectives, UFM activities turn out to be embedded in contrasting and competing food security discourses and agendas, from footloose/globalised food systems driven by high-tech solutions to outspoken preferences for re-localising food supply through social innovation and mediated by local landscapes. It reflects the vulnerability of the Dutch UFM as well as the multiplicity of food-security related reassembling in urban contexts (see also Cretella and Buenger 2016).

**Fig. 1:**
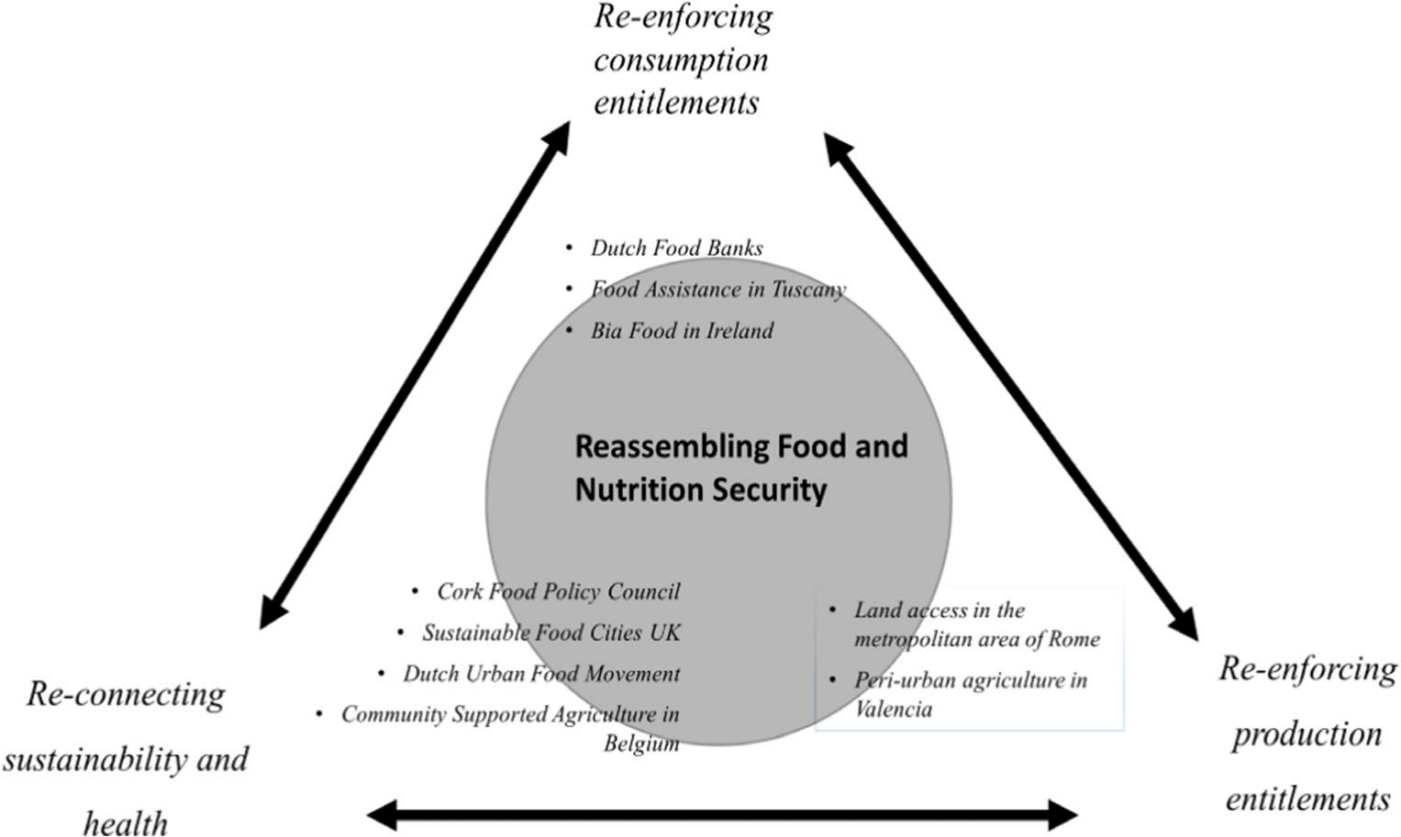
Reassembling food and nutritional security

From an assemblage perspective, it is important to note that this very multiplicity demonstrates how our reassembling categories should not be perceived as tightly defined, but much more as (partly) overlapping and interdependent sets of activities, practices and discourses (see also Fig. 1). For instance, *production*-entitlements oriented reassembling, though social and political, may simultaneously draw heavily on agro-ecologically inspired food quality motivations, thus on food *consumption* as another crucial reassembling component. Both land-access movements in Valencia and Rome indeed claim that place-based returns to strong rural-urban relations may positively contribute to healthy dietary behaviours and wider maintenance of food systems and urban lifestyles. Thus their reassembling turns out to be rooted in wider claims on integrative FNS and societal transformation, not just in *production* entitlements.

Ongoing reassembling centring on *food assistance* discloses particularly how its combination with wider sustainability–health issues may be more or less successful. As stressed by food assistance practitioners in the Netherlands and Ireland, they often cannot afford to be critical in terms of nutritional value or food origin. Here food assistance turn outs to be primarily entangled with (or sometimes even competing with) food waste reduction as the principle sustainability component. So far only incidental, as in the case of Dutch Food Banks, novel interlinkages may nonetheless be established to complement available food assortments with some fresh produce. Italian ‘Emporia of Solidarity’ is clearly more successful in this respect. It succeeds in mobilising surplus provisions from regional producers of fresh fruit and vegetables – an achievement partly explained by Common Agricultural Policy (CAP)-related institutional change and support. As such, its reassembling addresses (temporary) food poverty needs of individuals and families more adequately, not only by providing fresh produce, but also by interlinking food assistance with food education. Again, it shows how ongoing food and nutrient security reassembling throughout Europe addresses its multifaceted nature rather differently and with highly place-specific features and dynamics.

This same reassembling underpins the role, intentions, ambitions and hopes of citizen-consumers. Belgian Community Supported Agriculture demonstrates how citizen-consumer commitment may be motivated by, on the one hand, the wish to sustain production methods as a prerequisite for healthy food consumption, and trust building through close and direct relations with local smallholder food producers, on the other. The Dutch Urban Food Movement exemplifies a plethora of urban actor-led attempts to reconnect sustainability and health concerns. The Cork Food Council, and especially the Sustainable Food City Network in the UK, show how urban administrations might start to respond proactively to such movements, increasingly also with support from corporate and social enterprises. Sometimes these emerging urban multi-actor alliances also take food poverty alleviation actively into account, as especially illustrated by Irish and English reassembling practices, including associated upscaling and institutional embedding attempts.

## Significance for food and nutritional security governance

Previous reassembling analysis tells us that food system changes are increasingly the outcome of complex governance arrangements encompassing not just state actors (by way of public policy and the paradigms that inform such policy), but also a broad spectrum of other relevant actors such as churches, social movements and corporate groups from the food regime. As such, our set of reassembling practices covers a range of responses to and deviations from primarily public and/or food regime actor-led change. Most, if not all, distance themselves from dominant routines and rhetoric that solving the food questions of our time is primarily a matter of science, markets or public policy-making, where the role of social actors and their everyday life experiences remain largely absent or ignored. This, in the social science literature, is understood as ‘rendering technical’ (Li 2007) or ‘Solution-Fix’ (Umans and Arce 2014). In contrast, ongoing reassembling as depicted in this article makes it possible to underline the significance of a range of different practices, routines and directions, including the reshuffling of responsibilities between public–private and civil actors. Put differently, our set of reassembling practices is by definition an integral part of novel governance arrangements. Some of these arrangements span innovative public–private and public–civil partnerships; others try to remain far away from the public and administrative domain. Food banks, peri-urban agriculture projects, community supported agriculture, are all dealing in their particular ways with the (re)organisation of material environments and a variety of governance components such as spatial zoning, means of transport, public health regulations, food baskets, charity, solidarity and commitment.

While our analysis has shown how food actors are actively involved in reassembling their food systems and generating varying degrees of success, food governance scholars have more recently instead and in contrast stressed the *wickedness* of the contemporary food security problematic (Ingram 2011; Candel 2014). They underline that food security debates involve a multitude of stakeholders with often different FNS frames in terms of the principle causes of food insecurity. In their analysis, they tend to add food security dimensions (Carolan 2013a; Allen 2013), which only increases the cacophony of competing discourses. In recent attempts to further unravel this wickedness, food governance scholars such as Candel (2014) identified and distinguished five challenges for European food governance: responsiveness, reflexivity, revitalisation, rescaling and resilience. Such a food governance framing raises all kinds of critical questions when applied to the distributive, composite and multi-logics agency characteristic of assemblages (McFarlane 2009). As our analysis has shown, some actors combat food poverty for normative and ideological reasons whereas others accept food poverty as a reality and an outcome of the neo-liberal turn in state policies or as a corporate social responsibility. Thus, reflexivity may focus on challenging the power relations within the food regime as well as, albeit more or less consciously and intentionally, reproduce dominant regime actors’ positions and interests. Similar ambiguous meanings emerge around other distinguished key challenges. In a world of continuous assembling and reassembling, qualifications such as ‘*resilient’,* ‘*responsive’ and ‘revitalizing’* are by their very nature controversial and problematic. Partly by introducing assemblage-thinking inspired notions as citizen-consumers (MacRae et al. 2012; Spaargaren and Martens 2005), prosumers (Ritzer 2014) or public-private partnerships (Kraak et al. 2011), this has been earlier noticed by food scholars that unravel and characterize alternative food networks (see also Dwiartama and Piatti 2016; Le Velly and Dufeu 2016; Phillips 2016; Rocha and Lessa 2009; Wiskerke 2009). Yet, our characterisation of FNS reassembling aspires to go a step further by surpassing dichotomies as ‘alternative’ versus ‘dominant’, ‘public’ versus ‘civic’ or ‘micro’ versus ‘macro’. Urban food initiatives may be rather differently interwoven with European re-allocation of agricultural subsidy flows, as has been illustrated by Italian and Dutch reassembling practices. Similar meaningful differences have been identified regarding public administrative entanglements with public-choice oriented reassembling practices in the UK and Latvia. Or regarding the specificity of the institutional and rural-urban relations of Spanish and Italian land-access movements, including their ability to mobilize support from global agro-ecological movements to overcome specific institutional barriers. These type of place specific relations, interdependencies and contingencies make it more appropriate to speak of ‘the politics of scale’, where, according to Wald and Hill (2016: 205) ‘*the movement of food across the world and the conditions under which it grows [..] is itself a consequence of scalar contestation’*. Candel’s (2014) undertheorizing of the significance of power relations and differences in normative and ideological positioning in relation to rescaling as well other identified principle FNS governance challenges, appears also in food scholars’ requests for more ‘joined-up’, ‘coherent’, ‘consistent’, ‘holistic’ or ‘integrative’ policy-making (Duncan 2015; Kirwan et al. 2017; Duncan and Barling 2012; Clapp and Murphy 2013). In line with such requests, Sassen (2008), analysing global policy processes from a ‘weak’ assemblage perspective (that is, by primarily making use of its dictionary meaning), comes to the conclusion that the world nowadays faces the challenge to facilitate ‘*the formation of larger and more encompassing normative orders*’ (ibid. 75). Apart from our welcoming of encompassing normative orders, we would argue that this way of envisioning future food governance contrasts rather sharply with the multiplicity of ongoing FNS reassembling. A ‘stronger’ assemblage-theory inspired approach allows us, in analytically much better ways, to emphasise, problematise and unpack the nature, features, roles, interaction, interwovenness and complexity of the power relations involved, and the variety in normative orders that are part of the wider processes of change and transformation characteristic of contemporary FNS governance.

## Conclusions

This paper has argued that an assemblage perspective offers added value for a critical analysis of contemporary FNS issues and the debate on how to address FNS problems. Novel and promising manifestations of FNS reassembling in a variety of spatiotemporal settings were analysed. We highlighted reassemblings as critical responses to dominant public-policy intervention logics, building instead new alliances, coalitions and also partnerships between public, private and civic actors. In so doing we found and investigated new forms of chain-based and cross-sectoral forms of cooperation across historical, cultural and socio-economic backgrounds. These assemblages unfold as good examples of ‘spaces of engagement and contestation’ (Marsden 2013, 2016) all of which exhibit a combination of ‘relative positive’, but perhaps ambiguous, processes of reassembling that do not *yet* lead to new assemblages, and ‘absolute positive’ processes of reassembling that *do* create new assemblages. These have come to form an integral component of the contemporary FNS landscape, and unfold next to and sometimes in interaction with those that are shaped by actors from the predominant food regime. In addition to unravelling the emergent character of these ‘alternative’ assemblages, their multiplicity and indeterminacy, an assemblage perspective helps to distinguish between and amongst FNS reassembling practices. The analysis needs to take account of the conditions in which they unfold by contextualising and situating their development in societal debates and concerns, and also fully account for local actors’ experiences with their reassembling of parts of their food systems. We believe it is important to comprehensively compare the growing diversity of solutions to food problems that are driven by FNS concerns which exhibit governance arrangements that Carolan (2013a, b, 2016) theorises as change based on ‘doing’, ‘enacting’ and ‘feeling’ or – more generally –‘thinking differently by doing differently’. It leads us to believe that an assemblage perspective may contribute to overcoming the ‘locked in’ nature of ‘rendering technical’ approaches to the central food questions of our times, and to shed more comprehensive light on the nature, complexity and principal challenges of associated governance processes. Such analysis demands new methodological avenues of exploration. Moreover, next to moving beyond dichotomy-based thinking and problematizing fashionable key notions, we see our attempts to elaborate and advocate a ‘stronger’ assemblage lens as the principle contribution to the ongoing FNS and food governance debate.

## Footnotes

1 The corporate food system is well documented and critically reviewed by Clapp (2016), van der Ploeg (2010), Patel (2007), Marsden (2003), George (1979) and many others.

2 The arguments are well presented in the sustainable intensification literature. See for instance Godfray et al. (2010), Tilman et al. (2011), Garnett et al. (2013) and particularly Fresco (n.d.). Not surprisingly, perhaps, these papers do not disagree with the ‘perfect storm’ positions elaborated earlier in this article.
